# Neuroplastic Correlates in the mPFC Underlying the Impairment of Stress-Coping Ability and Cognitive Flexibility in Adult Rats Exposed to Chronic Mild Stress during Adolescence

**DOI:** 10.1155/2017/9382797

**Published:** 2017-01-15

**Authors:** Yu Zhang, Feng Shao, Qiong Wang, Xi Xie, Weiwen Wang

**Affiliations:** ^1^CAS Key Laboratory of Mental Health, Institute of Psychology, Beijing, China; ^2^The University of Chinese Academy of Sciences, Beijing, China; ^3^School of Nursing, Binzhou Medical University, Yantai, China; ^4^School of Psychological and Cognitive Sciences, Beijing Key Laboratory of Behavior and Mental Health, Peking University, Beijing, China

## Abstract

Using a valid chronic mild stress (CMS) model of depression, we found that adolescent (postnatal days [PND] 28–41) CMS induced transient alterations in anhedonia that did not persist into adulthood after a 3-week recovery period. Previously stressed adult rats exhibited more immobility/despair behaviors in the forced swimming test and a greater number of trials to reach criterion in the set-shifting task, suggesting the impaired ability to cope with stressors and the cognitive flexibility that allows adaptation to dynamic environments during adulthood. In addition, adult rat exposure to adolescent CMS had a relatively inhibited activation in ERK signaling and downstream protein expression of phosphorylated cAMP-response element-binding protein (CREB) and brain-derived neurotrophic factor (BDNF) in the medial prefrontal cortex. Further correlation analysis demonstrated that immobility and set-shifting performance were positively correlated with the inhibition of ERK signaling. These results indicated adolescent CMS can be used as an effective stressor to model an increased predisposition to adult depression.

## 1. Introduction

High plasticity is a fundamental mechanism of brain function for adaptation to dynamic environments. A large body of evidence has demonstrated that neuroplasticity in limbic areas is disrupted in depression, and antidepressant treatment produces therapeutic action by enhancing neuroplasticity [1]. For example, there exists a substantial decrease in the structural and functional plasticity in the prefrontal cortex (PFC) and hippocampus in depression; chronic treatment with antidepressants can reverse this decrease in parallel with the improvement of emotional symptoms [2, 3].

Early adverse experiences significantly increase the incidence of depression during adulthood [4, 5]. However, how stressor exposure during development causes long-term behavioral and physiological consequences is still not fully elucidated. The PFC is a critical brain region involved in the modulation of higher brain functions, including emotion, working memory, and cognitive function [6, 7]. In adolescence (conservatively estimated as the period from postnatal days [PND] 28 to 48 in rodents), the hypothalamic-pituitary-adrenal (HPA) axis is still immature and the PFC undergoes significant and profound development [8]. By causing enhanced and prolonged responses, stressor exposure during this stage may contribute to greater long-term detrimental effects in the development of these systems in adolescents than that in adults [9, 10]. Studies on humans and animals have consistently demonstrated that adverse stressors during adolescence lead to neuroplastic damage, including PFC volume loss, increased neuronal apoptosis, decreased neurogenesis, decreased synaptic transmission, and subsequently emotional and cognitive dysfunctions [11–14].

Extracellular signal-regulated kinase (ERK), one of the most important members of the mitogen-activated protein kinase (MAPK) family, is highly expressed in the PFC and hippocampus [15]. The activated ERK, phosphorylated ERK (pERK), subsequently modulates downstream nuclear transcription factors, especially cAMP-response element-binding (CREB) protein, which is involved in the transcription of several neurotrophic factors, such as brain-derived neurotrophic factor (BDNF). ERK-CREB signaling exerts extensive effects on behavioral and biological responses to stressors, such as synaptic plasticity, learning and memory, and emotion and cognitive function [16–19]. ERK signaling abnormality exists in the PFC of depressed patients and animals and can be improved by antidepressant treatment [20]. For example, our previous studies indicated that inhibition of ERK signaling in the mPFC and hippocampus was correlated with depressive behaviors induced by chronic cold water swimming stress, and treatment with antidepressant reversed these alterations [21, 22].

Chronic mild stress (CMS), consisting of multiple stressors that are analogous to the continuous and unpredictable life events in humans, is a valid rodent model of depression [23]. In adult rodents, CMS can induce a variety of behavioral and neurobiological changes, including anhedonia, decreased exploratory behavior, and increased immobility/despair behavior when exposed to stressful environments, impaired spatial cognition, and altered expression of ERK, CREB, and BDNF in the PFC [24–26]. Commonly, a longer time (usually 4–6 weeks or longer) is needed to establish this model, and its effects are transient and reversible shortly after the cessation of the stress. A few studies have investigated the long-term effects of CMS delivered during a specific time window, such as adolescence, and the results were inconsistent [12]. For example, Toth et al. reported that CMS from adolescence to early adulthood (PND 30–58) decreased anhedonia when tested 6 weeks after the stress [27], while Pohl et al. reported that CMS during adolescence (PND 23–51, followed by a 3-week no-stress period) increased anhedonia in adult female but not male rats [28]. In addition, predictable and unpredictable CMS during adolescence had protective and detrimental effects, respectively, on emotional and cognitive function in adulthood [29–31]. Thus the long-term effects of stress during adolescence on emotion and cognition are as yet not well characterized.

This study involved a set of behavioral tests to further evaluate the emotional and cognitive profiles of adult rats exposed to CMS solely during adolescence. Instinctive motivations for reward consumption and for exploration were determined, with decreases generally considered typical depressive symptoms. The forced swimming test (FST) was used to assess whether stress-coping ability to subsequent stressors during adulthood was affected by adolescent stress [9, 32, 33]. In addition, an attentional set-shifting task (AST) was performed to evaluate cognitive flexibility, a higher cognitive function that allows adaptation to a dynamic environment for optimized behavioral strategies. Strong evidence suggests that cortically mediated cognitive inflexibility is a core component of depression and is closely linked with attentional bias to negative emotion in depression, efficacy of antidepressant treatment, and recurrence of depression [34–36]. Additionally, the ERK-CREB signaling and downstream BDNF expression in the mPFC, a region involved in stress coping, emotion, and cognitive function, were determined, and the correlations between behavioral and molecular alterations were further analyzed. A dependence of these behavioral and molecular consequences on previous exposure to CMS during adolescence would suggest that exposure to this stressor at this stage can be an effective model to simulate the increased vulnerability to depression in adulthood by reducing neuroplasticity within the mPFC.

## 2. Methods and Materials

### 2.1. Animals

**Figure 1:**
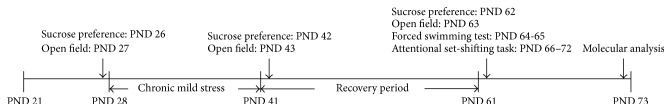
Timeline of procedures. We evaluated the effects of chronic mild stress during adolescence on adult behaviors using the following tests: sucrose preference, open field, forced swimming, and attentional set-shifting task.

Eighteen male Wistar rats were obtained from the Academy of Chinese Military Medical Science (Beijing, China) after weaning (PND 21). Rats were housed individually in stainless steel wire cages and were given 7 days to acclimate to the standard rearing conditions: 12 h light/12 h dark cycle (lights on at 7:00 am), ambient temperature of 20–22°C, and relative humidity of 40–70% with free access to food and water except during the sucrose preference test and the AST. A full timeline of all manipulations and behavioral tests is provided in Figure 1. All experimental procedures were approved by the Institutional Review Board of the Institute of Psychology, Chinese Academy of Sciences, and were in compliance with the National Institutes of Health Guide for the Care and Use of Laboratory Animals.

### 2.2. Stress Procedure

At PND 28, on the basis of a sucrose preference test, rats were divided into two matched groups and placed in separate rooms: a chronic mild stress (CMS) group (*n* = 9) and a control (CON) group (*n* = 9). Adapted from our previous study [37], the CMS procedure consisted of a variety of unpredictable mild stressors, including 12–24 h food and/or water deprivation followed in some cases by food restriction, cage tilt, continuous lighting, disrupted light/dark cycle, intermittent white noise, wet bedding, paired housing, flashing light (180 times/min), empty bottle stimulation, and hot or cold air (37°C for 30 min or 8°C for 30 min). Rats in the stress group were randomly exposed to 2–4 stressors every day for 2 weeks (PND 28–41). The controls were left undisturbed under the previously described maintenance conditions. Then, all animals experienced a 3-week no-stress period until adulthood. A series of behavioral tests were performed in sequence as follows to decrease any carry-over effects from one test to the other as much as possible.

### 2.3. Behavioral Tests

#### 2.3.1. Sucrose Preference Test

This test was performed before, one day after, and three weeks after the CMS exposure (PND 26, 42, and 62, resp.). Briefly, after 20 h of food and water deprivation, rats were offered two bottles containing either tap water or 1.5% sucrose solution. The liquid intake from each bottle was calculated by comparing the differences in bottle weights before and after a 1-h testing window. The sucrose preference was determined as the percentage of sucrose solution intake/total (water + sucrose liquid) intake. Low sucrose preference represented anhedonia, a core symptom of depression. When the preference test ended, rats were given free access to water.

#### 2.3.2. Open Field Test

This test was performed one day after each sucrose preference test (PND 27, 43, and 63). The testing apparatus was a circular arena (diameter × height: 180 cm × 50 cm). Rats were placed individually into the center of the arena and were recorded for 5 min. Exploratory behaviors, including horizontal ambulation and rearing, were determined. The distance of horizontal ambulation was automatically recorded using a computer-based tracking system (Med Associates Inc., USA), while the number of rearing instances was recorded by the experimenter and verified through video recording. The arena was thoroughly cleaned with 75% ethanol between each test to avoid any possible olfactory cues.

#### 2.3.3. Forced Swimming Test

The test was performed one day after the last open field test (PND 64 and 65). The paradigm was similar to that described elsewhere [38]. On the first day, rats were forced to swim in a glass cylinder with water (depth no less than 30 cm, 23-24°C) for 15 min. Then, the rats were dried and transported back to their home cages. On the second day, the rats were allowed to swim for 5 min. The 5-min test session was videotaped from above using a Sony Camcorder. Behavioral analysis proceeded as described in our previous study [39]. Briefly, the immobility time of each rat was measured with a stopwatch by a trained observer who was blind to the experimental treatments. Immobility was defined as a floating state in the water without struggling and making only those movements necessary to keep the head above water.

#### 2.3.4. Attentional Set-Shifting Task

This test was performed for one week from PND 66. The testing apparatus and procedure were introduced in our previous studies [13]. Briefly, rats were restricted to 10–14 g food per day to maintain 80–85% of their original body weight, with free access to water. Rats were trained to obtain a reward (1/4 Honey Nut Cheerios) by digging in two terracotta pots, which were defined by a pair of cues along two stimulus dimensions: the digging medium filling the pots and the odor applied to the inner rim of the pots. The “positive” pot was baited with a reward buried at the bottom of the digging medium. The test contained five successive stages with increased difficulty: the first stage was simple discrimination (SD), which only presented one relevant stimulus dimension (e.g., medium). The second stage was compound discrimination (CD) in which the same relevant stimulus dimension (medium) in SD was required and the second irrelevant dimension (e.g., odor) was presented as a distractor. The third stage was intradimensional shifting (IDS), wherein the medium was still the relevant dimension and the odor was still irrelevant, but new media and new odors were introduced. The fourth stage was reversal learning (RL), in which the same media and odors were used and the relevant dimension (medium) remained, but the negative cue in the IDS stage turned into a positive cue and the positive cue turned into a negative cue. The fifth stage was extradimensional shifting (EDS), in which all new media and odors were again introduced and the relevant dimension was replaced by odor. The test proceeded to the next stage when the rat reached a criterion of six consecutively correct trials. The number of trials to reach the criterion for each stage was recorded.

### 2.4. Western Blot Analysis

#### 2.4.1. Tissue Collection

The rats were decapitated one day after the behavioral tests. The whole brain was quickly removed, immediately frozen in liquid nitrogen and then stored at −80°C before tissue collection. The tissue collection method was analogous to that in our previous study [40]. In short, according to the rat brain atlas [41], the mPFC (3.20–2.20 mm from the bregma) was bilaterally punched using a stainless steel cannula with an inner diameter of 0.6 mm at −20°C in a cryostat microtome (Leica, CM 3050, Germany).

#### 2.4.2. Western Blotting Analysis

The tissue samples were placed in 50–70 *μ*L precooling lysate buffer (4°C, pH 7.5, containing 5 *μ*g/mL leupeptin, 5 *μ*g/mL aprotinin, 5 *μ*g/mL pepsin inhibitor, 5 *μ*g/mL trypsin inhibitor, 2 mM EDTA, 2 mM EGTA, 1 mM DTT, and 0.5% NP-40) according to their volume and then homogenized using an ultrasonic homogenizer (Sonic Co., Stratford, CT, USA). The protein concentrations in the homogenates were determined by a bicinchoninic acid (BCA) Protein Assay Kit (CW Biotech, Beijing, China). The homogenates were then mixed with 5x sodium dodecyl sulfate (SDS) in proportion to prepare sample solutions with a certain concentration. The prepared sample solutions were denatured at 95°C for 8 min. Denatured proteins (32 *μ*g) were separated by 12% SDS-PAGE and transferred onto a nitrocellulose (NC) membrane at 230 mA for 1 h. The membrane was blocked with 5% nonfat milk diluted in TBST overnight at 4°C. After being washed in TBST (10 min × 3), the membrane was incubated at RT for 2 h on a shaker with primary antibodies: rabbit monoclonal ERK1/2 antibodies (1 : 1,000, Cell Signaling Technology Inc., Beverly, MA, USA) and rabbit monoclonal pERK1/2 antibodies (1 : 2,000, Cell Signaling Technology Inc.). After further washing in TBST (10 min × 3), the membrane was incubated at RT for 1 h on a shaker with secondary antibodies, HRP-conjugated goat anti-rabbit IgG (1 : 4,000, Zhongshan Golden Bridge Biotechnology, Beijing, China), and then washed again. Bands were detected by enhanced chemiluminescence (ECL, Millipore, Bedford, MA, USA) through a FluorChem E System (ProteinSimple, Santa Clara, CA, USA). After exposure, the membrane was stripped and reprobed with primary mouse monoclonal GAPDH antibodies (1 : 1,000, Zhongshan Golden Bridge Biotechnology) and secondary HRP-conjugated goat anti-mouse IgG (1 : 4,000, Zhongshan Golden Bridge Biotechnology) following the above steps. The pCREB and BDNF levels and the corresponding GAPDH level were determined in a different NC membrane using the same procedures. All bands were quantified using Lab Works TM 4.6 (image acquisition and analysis software). The ratio of each target band intensity to the GAPDH band intensity was used for difference analysis between the CON and CMS groups.

### 2.5. Data Analysis

All data were presented as the mean ± standard error of the mean (M ± SEM). SPSS 19.0 was employed for statistical analysis. The body weight data, sucrose preference test results, open field test results, and attentional set-shifting task results were analyzed with a mixed model ANOVA with repeated measures (with stress as the between-subjects and time as the within-subjects variable). The remaining behavioral and molecular data were analyzed with a* t*-test for independent samples. For correlation analyses of mPFC molecular levels and behavioral alterations, Pearson's correlation analysis was adopted. The level of significance for all analyses was set at *p* < 0.05.

## 3. Results 

### 3.1. Effects of Adolescent CMS on Body Weights

**Figure 2:**
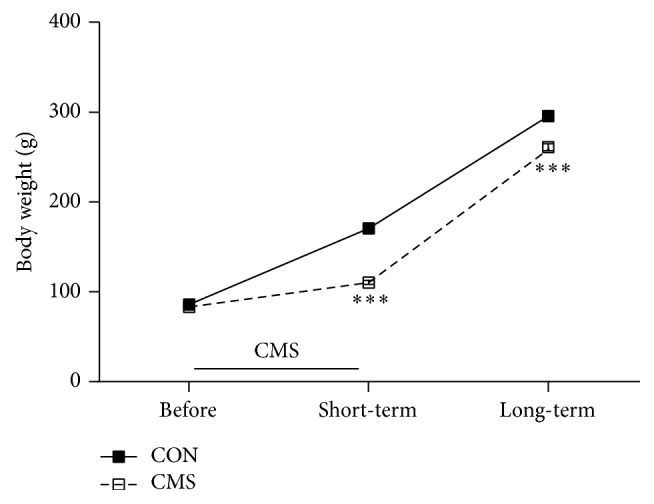
Effects of adolescent CMS on body weight. The body weights were measured on PND 28 (before the initiation of CMS), PND 42 (1 day after the end of CMS, short-term effect), and PND 63 (3 weeks after the end of CMS, long-term effect). ^*∗∗∗*^*p* < 0.001 compared to the CON group at the corresponding time points.

As shown in Figure 2, there were significant main effects for time point (*F*_(2,32)_ = 3075.488, *p* < 0.001) and stress (*F*_(1,16)_ = 78.249, *p* < 0.001) and a significant time point × stress interaction effect (*F*_(2,32)_ = 64.527, *p* < 0.001). Rats that were exposed to CMS during adolescence exhibited lower body weights than the corresponding controls when tested both immediately after the last stressor (*t*_16_ = 273.336, *p* < 0.001) and after a 3-week recovery period (*t*_16_ = 25.533, *p* < 0.001).

### 3.2. Effects of Adolescent CMS on Depressive Behaviors

**Figure 3:**
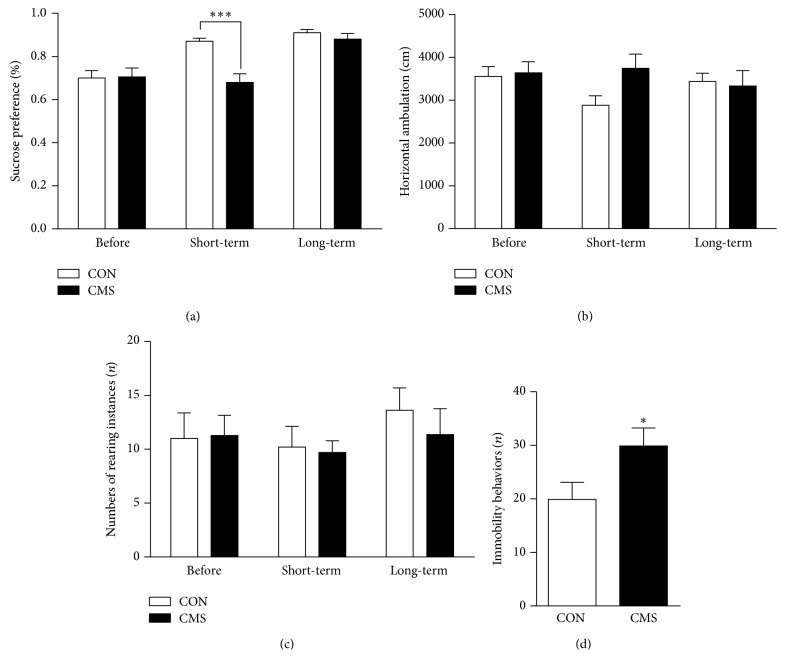
Effects of adolescent CMS on sucrose preference (a), horizontal ambulation (b) and number of rearing instances (c) in the open field test and immobility in the FST (d). Behavioral tests were conducted in sequence as follows: the sucrose preference test was performed before, one day after, and 3 weeks after the end of CMS; the open field test was performed one day after each sucrose preference test; the FST was performed one day after the last open field test in adult rats. ^*∗*^*p* < 0.05 and ^*∗∗∗*^*p* < 0.001 compared to the CON group at the corresponding time point.

For the sucrose preference test, there were significant main effects for time point (*F*_(2,32)_ = 19.893, *p* < 0.001) and stress (*F*_(1,16)_ = 6.495, *p* = 0.023) and a marginally significant time point × stress interaction effect (*F*_(2,32)_ = 3.154, *p* = 0.058; Figure 3(a)). The sucrose preference values were significantly lower in CMS-treated rats than in the corresponding controls one day after the end of CMS (*p* < 0.01), but this decrease did not persist into adulthood after the 3-week recovery period.

In the open field test, there were no significant main effects for time point (*F*_(2,32)_ = 0.790, *p* = 0.462) or stress (*F*_(1,16)_ = 0.460, *p* = 0.507). Likewise, the time point × stress interaction effect (*F*_(2,32)_ = 1.982, *p* = 0.153) for horizontal ambulation was not significant (Figure 3(b)). Similarly, there were no significant main effects for time point (*F*_(2,32)_ = 0.479, *p* = 0.623) or stress (*F*_(1,16)_ = 0.804, *p* = 0.382) and no significant time point × stress interaction effect (*F*_(2,32)_ = 0.446, *p* = 0.513) for the frequency of rearing (Figure 3(c)).

When the FST was conducted during adulthood, CMS-treated adult rats exhibited significantly increased immobility/despair behaviors (*t*_16_ = −2.135, *p* = 0.049; Figure 3(d)).

### 3.3. Effects of Adolescent CMS on Cognitive Flexibility in the AST

**Figure 4:**
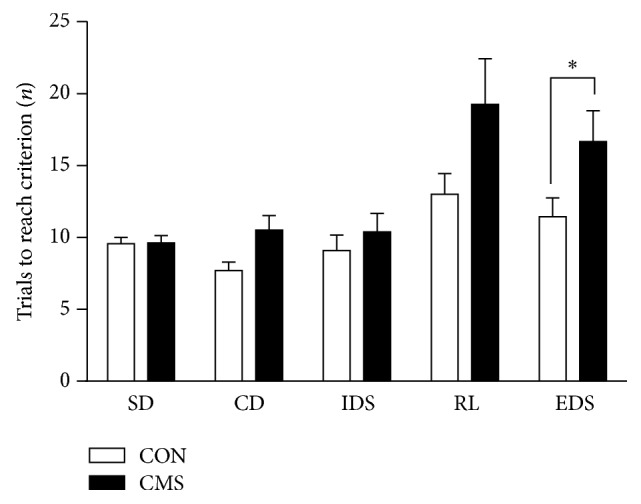
Effects of adolescent CMS on the performance on the AST. Rats were tested 3 weeks after the end of CMS during adulthood. The number of trials to criterion reflected the performance during the 5 stages of the test: simple discrimination (SD), compound discrimination (CD), intradimensional shifting (IDS), reversal learning (RL) and extradimensional set-shifting (EDS). ^*∗*^*p* < 0.05 compared to the CON group at the corresponding stages.

A two-way ANOVA revealed significant main effects for stage (*F*_(4,64)_ = 9.884, *p* < 0.001) and stress (*F*_(1,16)_ = 6.939, *p* = 0.022) but no significant stress × stage interaction effect (*F*_(4,64)_ = 1.905, *p* = 0.125). However, a* t*-test for each stage in the AST demonstrated a significantly greater number of trials to reach criterion in the EDS stages for the CMS-treated rats than for the controls (*t*_16_ = 4.970, *p* = 0.046). In addition, CMS-treated rats exhibited a moderately increased number of trials to criterion in the RL stages than the controls, but the difference was not significant (*t*_16_ = 3.463, *p* = 0.087) (Figure 4).

### 3.4. Effects of Adolescent CMS on ERK1/2, pERK1/2, pCREB and BDNF Levels in the mPFC of Adult Rats

**Figure 5:**
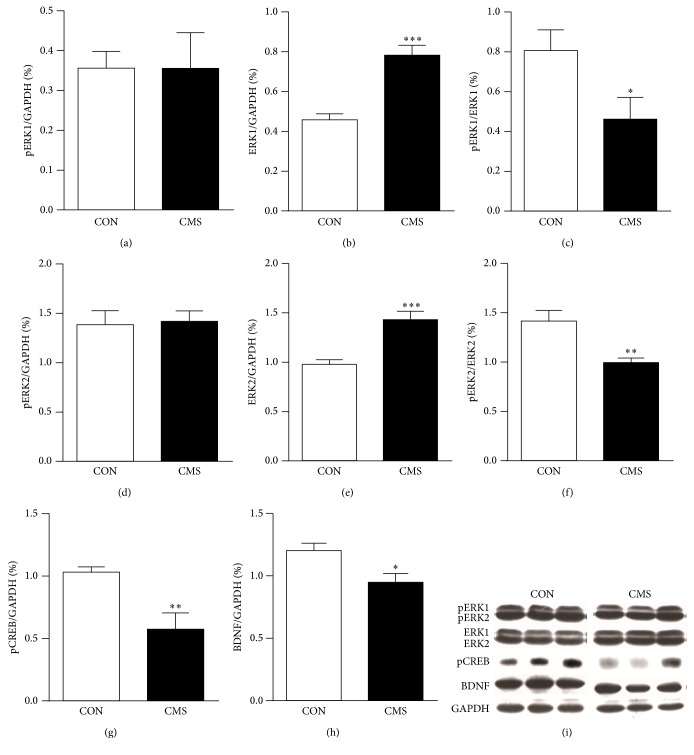
Effects of adolescent CMS on ERK1/2, pERK1/2, pCREB and BDNF levels in the mPFC of adult rats. (a) pERK1; (b) ERK1; (c) pERK1/ERK1; (d) pERK2; (e) ERK2; (f) pERK2/ERK2; (g) pCREB; (h) BDNF; and (i) representative blots for pERK1/2, ERK1/2, pCREB, BDNF and GAPDH. Rats were decapitated for western blot analysis one day after the behavioral tests. The results were calculated as the intensity of the lane of each transcript relative to the intensity of the corresponding GAPDH band and expressed as the mean ± SEM. ^*∗*^*p* < 0.05, ^*∗∗*^*p* < 0.01, ^*∗∗∗*^*p* < 0.001, compared to the CON group.

Adult rats exposed to adolescent CMS showed significantly increased expression of ERK1 (*t*_16_ = −6.087, *p* < 0.001) and ERK2 (*t*_16_ = −4.759, *p* = 0.001), but the pERK1 and pERK2 levels did not differ between the two groups (*p* > 0.05 for both; Figures 5(a), 5(b), 5(d), and 5(e)). Furthermore, the ratio of pERK1 to ERK1 (pERK1/ERK1, *t*_16_ = 2.290, *p* = 0.043) and the ratio of pERK2 to ERK2 (pERK2/ERK2, *t*_16_ = 3.435  *p* = 0.006) in CMS-treated rats were significantly lower than those of the CON group in adulthood (Figures 5(c) and 5(f)). The level of pCREB, one of the downstream nuclear transcription factors of ERK, and the level of BDNF were likewise decreased in adulthood (*t*_16_ = 3.550, *p* = 0.005 for pCREB; *t*_16_ = 2.855, *p* = 0.016 for BDNF; Figures 5(g) and 5(h)).

### 3.5. Correlations between Behavioral and Molecular Alterations in Adult Rats

As shown in Table 1, further correlation analysis demonstrated that the increased immobility behaviors in FST were positively associated with the increased ERK1 protein levels in the mPFC (*r* = 0.611, *p* = 0.026). There was a marginally negative correlation between the increased immobility behaviors and the decreased pERK2/ERK2 ratio (*r* = −0.535, *p* = 0.060). In addition, a marginally negative correlation was found between the increased trials to criterion in the EDS stage in AST and the decreased pERK1/ERK1 ratio (*r* = −0.557, *p* = 0.057).

## 4. Discussion

Adolescent CMS decreased body weight in rats in the current study, even after the 3-week recovery period and into adulthood, reflecting the stressful nature of this paradigm and its prolonged effect. We observed several novel findings in the present study: (1) Adolescent CMS increased immobility in the FST and impaired the set-shifting ability in adult rats even after several weeks of recovery. On the other hand, sucrose preference, while reduced immediately after the CMS, showed recovery and was normal in adulthood. Exploratory activity in the open field test was not affected by the stress exposure during adolescence. (2) Adolescent CMS decreased the relative activation of ERK signaling in the mPFC and the expression of the downstream pCREB and BDNF, which play important roles in brain development and neuroplasticity. (3) Adolescent CMS-induced behavioral alterations were correlated with ERK signaling activities in the mPFC. These results suggested that adolescent CMS did not affect instinctive motivation to consume a reward or exploration but significantly impaired the ability to cope with stressor exposure and cognitive flexibility that allows adaptation to dynamic environments during adulthood. The decreased neuroplasticity of the mPFC may mediate this procedure.

### 4.1. Effects of Adolescent CMS on the Behaviors of Adult Rats

First, adolescent CMS transiently induced anhedonia shortly after the stress, but this change was reversible, with recovery occurring 3 weeks later. Stress effects on anhedonia can be affected by some experimental factors, such as gender and stress conditions. For example, CMS through childhood and adolescence (PND 23–51) followed by a 3-week no-stress period caused a decrease in sucrose preference and an increase in anxiety behavior in a burying test in female but not male adults [28]. In addition, the unpredictability of the multiple stressors of the CMS presentation is also important because of its effects. Previous research showed that predictable and unpredictable stress during adolescence exerted protective and detrimental effects, respectively, on emotional and cognitive function in adult rats [29–31]. In this study, 2 to 4 types of stressors were randomly delivered at different times every day to maintain a continuous environmental disturbance. On this basis, we found that CMS that was limited to adolescence (PND 28–37) had no effect on the anhedonia of adult rats. Similar results were also observed in exploratory behaviors in the open field test. Fewer attempts to consume a reward and decreased exploration are thought of as typical depressive symptoms. Thus, these results suggested that adolescent CMS did not induce such signs of depression into adulthood.

Second, we found that adult rats with CMS experience during adolescence exhibited more immobility in the FST, suggesting that the animals exposed to adverse events during this period underwent a long-term change in the ability to cope with subsequent challenges during adulthood. The two-stage FST (the first stage was forced swimming training in an unescapable threatening environment; the second stage was a helpless or despair behavior test) has been extensively used to identify an inability to cope with subsequent stressors after excessively adverse experiences, a symptom of depression called helplessness/despair [42]. Thus, our results suggested that previously stressed rats had higher susceptibility to develop despair behavior in a stressful environment during adulthood. Similarly, other studies also showed that the changes induced by stress exposure during adolescence can be long-term but only become apparent after a subsequent stressor is applied, which may contribute to an increased risk for stress-related disorders later in life [9, 43, 44]. In addition, although lower body weight was found in previously stressed rats, there had been similar locomotor activity in open field test in control and stress groups, suggesting that the increased immobility in stress group was unlikely due to deficits in body energy.

Third, we found that adolescent CMS impaired the cognitive flexibility of adult rats, as indicated by the increased number of trials to reach criterion in the EDS stage. Cognitive inflexibility is increasingly recognized as a relatively independent risk factor for the onset of depression, which is closely associated with emotional inflexibility in depression, therapeutic effects on emotional symptoms after antidepressant treatment, and the reoccurrence of depression [34–36]. Normal function of the mPFC is necessary to perform set-shifting [7]. Chronic stress can impair set-shifting performance as a result of the structural and functional effects on the mPFC, which can be ameliorated by antidepressant treatment [45, 46]. Therefore, the impaired EDS performance demonstrated in this study may reflect mPFC dysfunction during adulthood.

### 4.2. Effects of Adolescent CMS on Neuroplastic Molecules in the mPFC of Adult Rats

We found that adolescent CMS induced an increase in the expression of ERK1 and ERK2 but not pERK1 and pERK2 in the mPFC of adult rats. Accordingly, the pERK1/ERK1 and pERK2/ERK2 ratios were decreased in previously stressed adult rats compared to controls. Phosphorylated ERK indicates an activated state of ERK, while the pERK1/ERK1 and pERK2/ERK2 ratios reflect the relative activation and actual effects on neural cells [47, 48]. These results suggested that adolescent CMS exerted inhibitory effects on ERK signaling in the mPFC. CREB, a main downstream nuclear transcription factor regulated by ERK signaling, can modulate the transcription of many proteins, such as c-fos and BDNF. The ERK-CREB cascade has been confirmed to be involved in the modulation of neural development, neuroplasticity, stress responses, emotion, and cognition [19, 49]. In line with the inhibition of ERK signaling, there was a long-term decrease in the pCREB and BDNF levels in the mPFC of adult rats subjected to adolescent CMS. Commonly, acute stress increases ERK signaling in the mPFC for adaptation, while chronic stress decreases this signaling and causes functional impairment [22, 47, 50, 51]. Animals with ERK or BDNF gene knockdown also exhibited structural and functional abnormalities in the mPFC [52, 53]. In addition, studies on humans and animals have shown that adolescent stress can cause long-term decreases in the neuroplasticity of the mPFC, which are accompanied by increased depression and anxiety during adulthood [11, 12]. These results suggest that the intracellular ERK-CREB cascade may mediate the decreased neuroplasticity of the mPFC induced by adolescent stress.

### 4.3. Relationship between Behavioral and Molecular Alterations in Adult Mice Exposed to CMS during Adolescence

Further correlation analysis showed that immobility in the FST was positively correlated with ERK1 protein levels, while there was a marginal negative correlation with the pERK2/ERK2 ratio; the number of trials to criterion in the EDS stage tended to negatively correlate with the pERK1/ERK1 ratio. In adult rats exposed to chronic cold water swimming stress, depressive behaviors including anhedonia and locomotor activity were correlated with changes in the pERK1 and pERK2 and the pERK1/ERK1 and pERK2/ERK2 ratios, which could be reversed by treatment with the antidepressant fluoxetine [22, 51]. Although these studies also suggest that inhibition of ERK signaling in the mPFC is linked with depression, the characteristics of these alterations are different between the two studies. The reasons remain unclear, but the developmental stage when stressors are applied may be an important factor. For example, the mPFC is necessary for juveniles (early stage of adolescence) to process threatening stimuli [54]. In addition, Bingham et al. [55] reported that social defeat during the early adolescent but not late adolescent and adult periods altered the activity of the noradrenergic system and its projecting area, the mPFC, suggesting that the mPFC is an area sensitive to stress during early adolescence, a stage that is similar to that in the present study. Notably, set-shifting performance is specifically regulated by noradrenaline in the mPFC and also involves ERK signaling [7, 56, 57]. For example, the noradrenaline-induced long-term inhibition of pyramidal neuron synapses depends on postsynaptic activation of ERK1 and ERK2 signaling [57]. Imipramine (an antidepressant that can increase brain noradrenaline levels) can improve set-shifting performance, and this effect can be blocked by an inhibitor of ERK1 and ERK2 signaling [58, 59].

## Figures and Tables

**Table 1:** Correlations between behavioral and molecular alterations in adult rats exposed to adolescent CMS.

		ERK1	ERK2	pERK1/ERK1	pERK2/ERK2	pCREB	BDNF
Immobility	*r* value	0.611	0.454	−0.338	−0.535	−0.468	−0.139
	*p* value	0.026^*∗*^	0.119	0.259	**0.060**	0.106	0.652

EDS	*r* value	0.142	−0.022	−0.557	−0.350	−0.101	−0.011
	*p* value	0.659	0.945	**0.057**	0.264	0.754	0.972

^*∗*^ *p* < 0.05.

## Additional Points

*Summary*. Our results indicate that CMS during adolescence induced long-term behavioral and molecular effects characterized by impairments in stress-coping ability and in cognitive flexibility required for adaptation to dynamic environments during adulthood. The results also indicate that these features may be mediated by decreased neuroplasticity in the mPFC. Thus, adolescent CMS can be used as an effective stressor to model an increased predisposition to adult depression to further elucidate the underlying neurobiological mechanisms of this condition.
